# Ubiquitin chain topology in plant cell signaling: a new facet to an evergreen story

**DOI:** 10.3389/fpls.2014.00122

**Published:** 2014-04-01

**Authors:** Charlotte K. Walsh, Ari Sadanandom

**Affiliations:** School of Biological and Biomedical Sciences, University of DurhamDurham, UK

**Keywords:** ubiquitin, plants, signaling pathways, abiotic stress, pathogen

## Abstract

Ubiquitin is a peptide modifier able to form polymers of varying length and linkage as part of a powerful signaling system. Perhaps the best-known aspect of this protein's function is as the driver of targeted protein degradation through the Ubiquitin Proteasome System (UPS). Through the formation of lysine 48-linked polyubiquitin chains, it is able to direct the degradation of tagged proteins by the 26S proteasome, indirectly controlling many processes within the cell. However, recent research has indicated that ubiquitin performs a multitude of other roles within the cell beyond protein degradation. It is able to form 6 other “atypical” linkages though lysine residues at positions 6, 11, 27, 29, 33, and 63. These atypical chains perform a range of diverse functions, including the regulation of iron uptake in response to perceived deficiency, repair of double stranded breaks in the DNA, and regulation of the auxin response through the non-proteasomal degradation of auxin efflux carrier protein PIN1. This review explores the role ubiquitin chain topology plays in plant cellular function. We aim to highlight the importance of these varying functions and the future challenges to be encountered within this field.

## Introduction

Posttranslational modification (PTM) is a process through which proteins are altered after ribosomal synthesis. This process includes the formation of disulfide bridges, the alteration of amino acids and the addition of new functional groups, thereby changing the proteins from their nascent state to one of full functionality, or changing the function of the mature proteins. Ubiquitin and ubiquitin-like proteins (UBLs) are perhaps the most well-known set of proteins involved in the latter type of PTM.

Ubiquitin is a 76 amino acid protein so named because of its ubiquitous nature. It is part of a powerful signaling system that regulates several processes, the most well-known of which being the degradation of cellular proteins by the 26S proteasome in the Ubiquitin/Proteasome System (UPS). However, the ubiquitin signaling system is versatile and is able to regulate not only the abundance of protein present within the cell by degradation, but it is also able to target proteins to particular organelles (such as the nucleus), initiate membrane receptor recycling, and recruit proteins in the DNA damage repair pathway. Its importance can be seen by the fact that over 6% of the proteins in the *Arabadopsis thaliana* proteome are part of the ubiquitin pathway (Downes and Vierstra, 2005).

Ubiquitin contains 7 lysine residues: K6, K11, K27, K29, K33, K48, and K63. Through these 7 lysine residues and its N-terminal methionine (M1) it is able to form polyubiquitin chains upon a target protein. This provides a huge scope for variation in linkages and thus allows several functions to be encoded by just one peptide tag.

## Making chains

The ubiquitination of a target protein occurs through an exposed lysine residue. The ε-amino group of the lysine forms a bond with ubiquitin through the carboxyl group of the C-terminal glycine (Pickart, 2004). This tag can then be extended, if required, into a polyubiquitin chain with the sequential ubiquitin moieties connected through lysine-glycine linked isopeptide bonds.

Four enzymes are required for ubiquitin conjugation of the tag to the target protein; a ubiquitin-activating enzyme (E1), a ubiquitin-conjugating enzyme (E2), and two ubiquitin ligases (E3 and E4). Together these enzymes form a cascade, with multiple rounds of repetition giving chain extension. To begin, the E1 activates the Ub moiety using ATP, forming a Ub-adenylate (Schulman and Harper, 2009), which is then bound by the E1. The activated Ub is then transferred to the E2, which correctly orientates the moiety, and the complex recruited by an E3 (Spratt et al., 2012), resulting in the transfer of Ub to the target protein. The chain is then subsequently extended through either E3 or E4 activity (Koegl et al., 1999).

## Lysine 48-linked polyubiquitin chains

Ubiquitin chains connected by “typical” K48-linkages perform one of the most well-known functions of ubiquitin—proteasome targeting. The UPS has been implicated in many aspects of plant function. As plants are sessile organisms, a greater degree of phenotypic plasticity is required to ensure survival in a changing environment. Plants must be able to respond quickly and efficiently to relevant stimuli and this is achieved, in part, by the targeted degradation of proteins by the proteasome.

The function of K48 chains and the UPS has been the subject of several excellent reviews (see Moon et al., 2004; Dreher and Callis, 2007). The UPS plays a major role in plant development, hormone signaling, pollen tube growth, pathogen defense, and the cell cycle.

One specific example is the involvement of the UPS in self-incompatibility (SI). SI is a mechanism by which flowering plants are able to avoid inbreeding due to self-fertilization. In *Brassicaceae sp*., the UPS plays a role in SI through the U-box-dependant E3 ligase ARM-repeat-containing 1 (ARC1), as shown by the breakdown of SI upon antisense downregulation (Stone et al., 1999). ARC1 is thought to ubiquitinate a compatibility factor in the pistle, leading to its degradation in the proteasome and the rejection of self-pollen (Stone et al., 2003).

Another example of K48-linked polyubiquitin chains and their role in proteasomal degradation is auxin-mediated SCF^TIR1/AFB^ (Skp1, Cullin, F-box receptor-type ubiquitin ligase) pathway. Auxin is a plant hormone that regulates gene expression with respect to plant development. It binds to TIR1 or AFB (F-box) components of the SCF^TIR1^ RING-type E3-ligase complex, enhancing the affinity of SCF^TIR1^ for Aux/IAA transcription regulator proteins. Aux/IAA proteins, such as SHY2 and BDL, form heterodimers with ARF (Auxin Response Factor) transcription factors, resulting in repression of genes controlled by auxin-responsive elements (AuxREs). The SCF^TIR1^ polyubiquitinates these Aux/IAAs and causes their destruction by the 26S proteasome (Maraschin et al., 2009). This results in the binding of ARFs and transcription of specific auxin response genes.

## Lysine 63-linked polyubiquitin chains

K63 polyubiquitin chains have been discovered in both single and multicellular eukaryotes and have been shown to regulate several processes in yeast and mammalian cells in a non-proteolytic manner, such as kinase activation, protein synthesis, DNA repair, and chromosome regulation (Jacobsen et al., 2009). Their function in plants, however, has been less well-characterized, with K63 chains only implicated in apical dominance, DNA repair and iron deficiency mechanisms to date (Li and Schmidt, 2010)

Auxin performs many roles within the plant, as seen above, one of which being apical dominance, where the main central stem exhibits dominance over side shoots. The mutation of two membrane-associated E3 ubiquitin ligases, RGLG1 and RGLG2, exhibits a loss of apical dominance due to reduced cellular auxin concentration and the inability to transcribe auxin-responsive genes upon the application of exogenous auxin (Yin et al., 2007). RGLG1 and RGLG2 have been shown to catalyze the formation of K63 ubiquitin chains *in vitro*, indicating a role for this type of ubiquitin linkage in the regulation of apical dominance.

Unlike the SCF-TIR1 pathway described earlier, RGLG1 and RGLG2 seem to play a role in regulating intracellular auxin levels through PIN (auxin efflux carrier protein) cycling. PIN2 has been shown to undergo K63-linked ubiquitination by RGLG2, with the chain acting as a signal for endocytosis and transport to the vacuole. Analysis of this pathway using an *rglg*^−^ mutant indicates that ubiquitinated PIN2 plays a role in root gravitropism (Leitner et al., 2012). PIN1 also appears to be a RGLG1/2 target. Evidence indicates that PIN1 abundance is reduced in *rglg1/rglg2* knock out mutants, and an interaction has been demonstrated between RGLG2 and PIN1 in yeast two-hybrid studies (Yin et al., 2007).

K63 linked polyubiquitin chains are also implicated in iron deficiency signaling. Experiments involving the ectopic expression of a cucumber Ubc13 homolog (CsUbc13) in *Arabidopsis* showed the production of bifuricated root hairs, a classical response seen in iron-deficient plants (Li and Schmidt, 2010). Further work by Li and Schmidt indicated an interaction between Ubc13 and RGLG2, with *rglg1/rglg2* double mutants showing constitutively active root hair bifurication, suggesting that auxin directs in morphological responses to iron deficiency (Nagpal et al., 2000).

## Lysine 29-linked polyubiquitin chains

Recent research (Wang et al., 2009) has revealed the role of K29-linked chains in the degradation of DELLA proteins. The DELLA protein family are a group of growth repressors involved in the gibberellic acid (GA) response (Fleet and Sun, 2005). In *Arabidopsis* there are five known DELLA proteins: GAI, RGA, RGL1, RGL2, and RGL3 (Cheng et al., 2004). Involvement of these proteins has been shown in several important environmental responses, such as the light, cold, and salt responses (Achard et al., 2007, 2008; Magome et al., 2008).

DELLA degradation, induced by GA, is an important part of this signaling pathway (Dill et al., 2001). In a cell free system, Wang et al. (2009) were able to show that K29-linked Ub chains were responsible for the targeting of DELLA proteins to the 26S Proteasome. This indicates that the K29 linkage provides a similar function to that of K48.

## Atypical linkages (K6, K11, K27, K33, and M1)

Unlike K48- and K63-linked chains, there has been very little research on other atypical polyubiquitin linkages, with none conducted in plant systems. Experiments in eukaryotes such as *S. cerevisiae* have shown the formation of chains liked via K6, K11, K27, and K33. Research has shown that these atypical linkages play a role in mammalian DNA repair (Wu et al., 2008), cell cycle control through the APC/C complex (Williamson et al., 2009), lysosomal localization of transcription factors (Ikeda and Kerppola, 2008), proteasomal degradation (Xu et al., 2009), and the regulation of signal transduction through the prevention of TCR-ζ and Zap-70 association (Huang et al., 2010), respectively. However, much of the function of these chains remains currently unknown.

As well as chains formed through lysine-glycine linkages, ubiquitin is also able to conjugate through the methionine residue located at the N-terminus (M1) (Emmerich et al., 2011). In doing so, it forms a linear chain which has been shown to play an important role in mammalian signaling pathways, such as those involving tumor necrosis factor (TNF) (Haas et al., 2009) and NF-κ B (Tokunaga et al., 2009). To date, there has been no evidence to suggest the formation of M1-type linear chains in *Arabidopsis*.

## Determining chain specificity

As can be seen from the examples above, ubiquitin signaling is a very versatile system. Through the alteration of chain topology, the Ub system forms a large part of many, vastly different biological pathways. It is through an integral part of the Ub conjugation pathway, the E2, that this attribute is conferred.

The E2s are a large family of proteins, present in all eukaryotes. They are characterized by both their ability to interact with E1s and E3s and the presence of the UBC motif. The UBC motif consists of a highly conserved catalytic fold of approximately 150–200 amino acids in length (Kim et al., 2004). In this fold sits the catalytic cysteine residue through which the Ub moiety is accepted (Mukhopadhyay and Reizman, 2007).

The E2 family is able to influence the construction of ubiquitin chains of a specific linkage (Ye and Rape, 2009). This includes linear homogenous chains of a single linkage type, heterologous chains (i.e., Ub and SUMO Aillet et al., 2012) and branched/mixed linkage chains. Studies within the field showed that E2s are able to synthesize ubiquitin chains of a particular linkage even whilst not in the presence of an E3 (Hass et al., 1991). Later research identified several E2s in yeast and human cells that appear to predominantly form ubiquitin chains of K48, K63, and K11 linkage; the identified E2s are ScCdc34, ScUBC13/MMS2, and HsUBE2S, respectively, with their mechanisms for preferred lysine selection differing greatly.

Cdc34 is an E2 from *Saccharomyces cerevisiae* that forms ubiquitin chains with predominantly K48 linkages (Petroski and Deshaies, 2005). The specificity for this linkage type appears to be conferred by the interaction between the acceptor Ub and an acidic loop present within the E2 (Li et al., 2007). This selection mechanism for correct Ub orientation differs from that of the predominantly K63-forming UBC13/MMS2 complex, where the active site cysteine residue forms a bond with the donor Ub moiety, with the complexed MMS2 non-covalently binds the acceptor Ub in an optimal position for K63 linkage (Eddins et al., 2006). It is currently unknown how HsUBE2S orientates Ub moieties to ensure K11-linkage.

In *Arabidopsis thaliana*, 41 E2s have been identified to date (Kraft et al., 2005). In the study conducted by Kraft et al. (2005), a phylogenetic analysis of the identified E2s was conducted. This analysis identified several plant homologs to ScUBC1, ScUBC7, ScUBC13, and ScMMS2. Later research into these homologs, detailed in Table 1, has confirmed the role of the E2 in chain topology, with different E2s constructing chains of a distinct linkage (either “typical” K48-linkages or K63-linkages) upon their varied targets.

**Table 1:** ***Arabidopsis* E2 function**.

**Gene**	**Locus**	**Homolog**	**Linkage type**	**Processes involved in**	**Referenced in**
AtUBC11	AT3G08690	ScUBC1	K48	Mediator of selective degradation of abnormal and short-lived proteins.	Kraft et al., 2005
				Expression in floral tissues.	
AtUBC27	AT5G50870	ScUBC1	Predicted K48	Expression in seeds, siliques, pistils, hypocotyls, and leaves.	Kraft et al., 2005
AtUBC28	AT1G64230	ScUBC1	Predicted K48	Expression in seeds, siliques, pistils, hypocotyls, and leaves.	Kraft et al., 2005
AtUBC7	AT5G59300	ScUBC7	K48	Able to ubiquitinate BrARC1 *in vitro*.	van Nocker et al., 1996; Stone et al., 2003; Kraft et al., 2005
AtUBC13	AT3G64460	ScUBC7	Predicted K48	Upregulated by syringolin.	van Nocker et al., 1996; Kraft et al., 2005
				Caution: confusion in publications between AtUBC13 and AtUBC35 (AtUBC13A).	
AtUBC14	AT3G55380	ScUBC7	Predicted K48	Upregulated in the G0 to S phase transition of the cell cycle.	Genschik et al., 1994; van Nocker et al., 1996; Kraft et al., 2005
AtUBC35 (AtUBC13A)	AT1G78870	ScUBC13	K63	Mediator of transcriptional activation of target genes.	Kraft et al., 2005; Wen et al., 2006; Li and Schmidt, 2010
				Involved in UV damage repair.	
				Involved in root development regulation in response to iron availability.	
AtUBC36	AT1G16890	ScUBC13	K63	Partial functional redundancy with AtUBC35	Kraft et al., 2005; Wen et al., 2006; Li and Schmidt, 2010
AtUEV1A (AtMMZ1)	AT1G23260	ScMMS2	K63	Forms heterodimer with AtUBC35 and AtUBC36.	Yin et al., 2007; Wen et al., 2008
				Possibly involved in cell cycle control and differentiation.	
AtUEV1B	AT1G70660	ScMMS2	K63	Forms heterodimer with AtUBC35 and AtUBC36.	Yin et al., 2007; Wen et al., 2008
				Possibly involved in cell cycle control and differentiation.	
				May be involved in DNA repair.	
AtUEV1C	AT2G36060	ScMMS2	K63	Forms heterodimer with AtUBC35 and AtUBC36. Possibly involved in cell cycle control and differentiation.	Yin et al., 2007; Wen et al., 2008
				May be involved in DNA repair.	
AtUEV1D	AT3G52560	ScMMS2	K63	Forms heterodimer with AtUBC35 and AtUBC36. Possibly involved in cell cycle control and differentiation.	Yin et al., 2007; Wen et al., 2008
				Involved in DNA repair.	

*Collated data of Arabidopsis thaliana homologs of yeast and human E2s, showing the predicted/observed linkage type for each E2 and the processes in which it is involved*.

**Figure 1:**
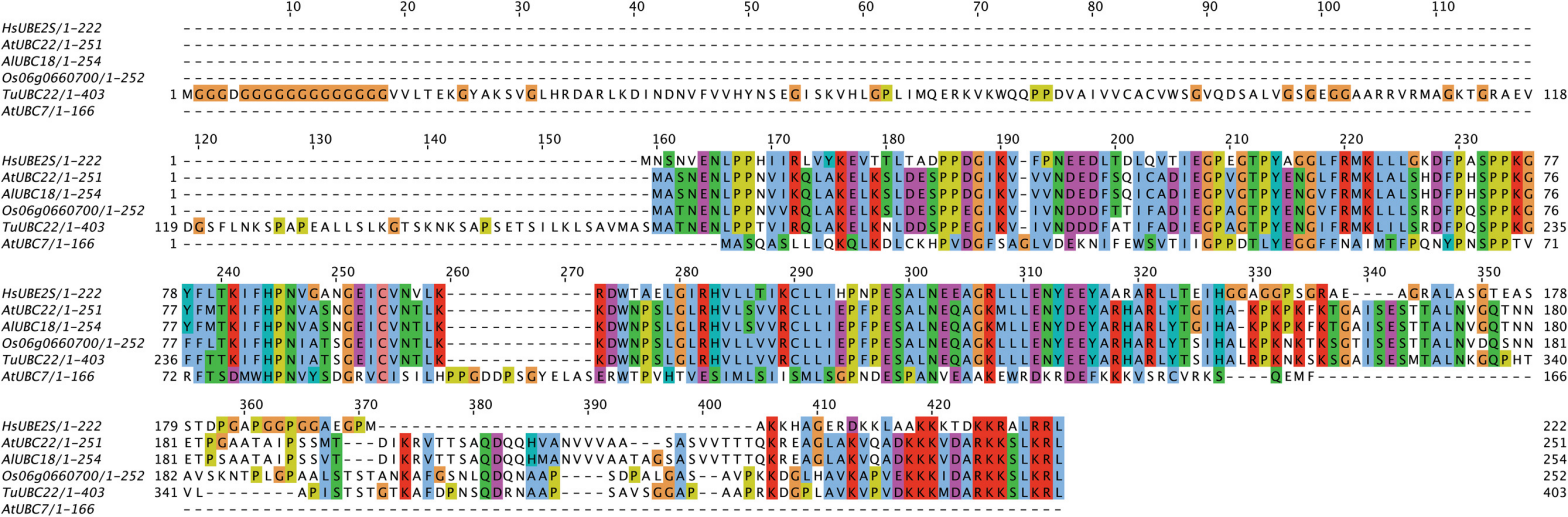
**Alignment of HsUBE2S with AtUBC22 homologs and AtUBC7**. An alignment showing the amino acid sequence similarity between the K11-chain-forming Human E2, UBE2S, the plant homologs AtUBC22, AlUBC18, Os06g0660700, and TuUBC22, and the K48-chain-forming AtUBC7. The alignment shows large areas of conservation amongst the K11-chain-forming E2s, but little in comparison with UBC7. The active site cysteine (red box) is conserved in all aligned sequences, with the surrounding residues showing very high conservation amongst the K11-chain-forming UBCs.

Aside from the E2s detailed in Table 1, the analysis of Kraft et al. (2005) also identified an *Arabidopsis* homolog to the K11-linkage forming HsUBE2S. This homolog, UBC22 (At5g05080), along with other homologs from *Arabidopsis lyrata* (AlUBC18), *Oryza sativa* (Os06g0660700), and *Triticum urartu* (TuUBC22) identified using BLAST (Altschul et al., 1997), showed large areas of conservation in the UBC fold as well as a conserved active site cysteine residue (Figure 1). This suggests that AtUBC22 and its homologs may also construct K11-linked chains as seen with HsUBE2S.

## Linkage analysis

One inherent obstacle to research into polyubiquitin chain topology is the method of analysis used. Currently there is no direct way of assessing linkages between ubiquitin moieties in a chain. Most information gathered in this area has been obtained through a combination of mutant ubiquitin usage and mass spectrometry, neither of which are without their flaws.

The use of mutant ubiquitin to study chain topology has a number of potential problems associated with it. Creating satisfactory controls for experiments using mutant ubiquitin *in vivo* are difficult. Overexpresson of mutant ubiquitin may alter which substrates are ubiquitinated, and by what type of chain. Also, the mutation of lysine residues to arginine to prevent conjugation may alter the surrounding surface of the protein. An example of this would be the alteration of the K6 residue. K6-sulfosuccinimidobiotin-labled ubiquitin is able to form polyubiquitin chains, but conjugates show a lesser susceptibility to proteasomal degradation. Mutation of the lysine residue (K6W) also gives a similar effect (Shang et al., 2005). This suggests that K6, or the surrounding surface environment, is required for proteasomal degradation through recognition and binding to the 26S proteasome.

Mass spectrometry is one of the most powerful tools available for studying changes in ubiquitin linkages. Often proteins of interest are purified using an affinity matrix, then their linkages analyzed by mass spectrometry. The typical method used involves the detection of a signature tryptic peptide. This peptide is derived from ubiquitin attached through its lysine residue to the—GG or—LRGG of another ubiquitin moiety (Saracco et al., 2009). However, the results may be skewed in favor of chains with a higher relative abundance, making the complexity of less abundant chains harder to determine.

Aside from the more commonly used forms of linkage identification detailed above, it is also possible to determine Ub chain type through the use of deubiquitinating enzymes (DUBs) that specifically cleave ubiquitin moieties from target proteins. Due to structural disparities between multiubiquitin chains of differing linkage, DUBs show binding-site specificity for particular linkages (Reyes-Turcu et al., 2009). For example, the DUB OTU7B shows specificity toward K11-linked chains (Bremm et al., 2010; McGouran et al., 2013). This specificity could be exploited to determine chain linkage *in vitro*.

## Conclusion

Ubiquitin is an effective peptide modifier that forms part of a powerful, if poorly understood, signaling system. By differing the selection of E1, E2, and E3s used in chain formation, polyubiquitin chains of differing linkage, and thus function, can be formed upon target proteins. The degree of variation of function achieved through these linkages creates a versatile system enabling sessile organisms, such as plants, a greater degree of phenotypic plasticity and thus the ability to adapt to a rapidly changing environment.

Further research into the function of atypical chains in plants is required. However, perhaps the biggest obstacle to overcome in terms of the determination of polyubiquitin linkage in plants is the inability to directly assess topology both *in vivo* and *in vitro*. The development of a direct method of linkage assessment would eliminate the use of mutant ubiquitin as the primary method of chain determination.

### Conflict of interest statement

The authors declare that the research was conducted in the absence of any commercial or financial relationships that could be construed as a potential conflict of interest.
